# Auditory noise influences human visual perception of ambiguous information: multi-modal integration during bistable perception

**DOI:** 10.1186/1471-2202-16-S1-P191

**Published:** 2015-12-18

**Authors:** Woochul Choi, Se-Bum Paik

**Affiliations:** 1 Department of Bio and Brain Engineering, KAIST, Daejeon 305-338, Republic of Korea

## 

When the sensory system receives an ambiguous signal, human perception often switches spontaneously between two different interpretations. This phenomenon is called bistable perception, and has been considered important to understanding sensory system. In this study, we investigated the intervals of spontaneous switching, defined as reversal time τ, to examine the temporal dynamics of bistable perception. We also studied the multi-modal feature of bistable switching by applying auditory noise with the visual stimuli. Our hypothesis is that auditory noise would significantly alter the reversal time of bistable visual perception. By building a computational model, we could explain the influence of auditory noise on the reversal time.

In the human psychophysical experiments, we first measured the reversal time with visual stimulus only, using two types of bistable visual movies: the racetrack [1] and the rotating 3D cylinder (Figure 1A). We observed that the reversal times are widely varied across the subjects but fairly consistent within the subject in both cases. Interestingly, we also found that the reversal time for the racetrack and the rotating cylinder were highly correlated (N = 9, R^2 ^= 0.84, Figure 1C). Next, we performed the experiment with auditory noise and visual stimuli together, and found that the reversal times are significantly altered. Importantly, when auditory noise was given, we found a systematic change such that a fast switching subjects (short τ) tend to slow down the switching while slow switching subjects (long τ) tend to speed up, so that the difference of τ between the two groups become insignificant (Figure 1D). Lastly, we designed a double-well energy model with destabilization/restabilization processes [2], and the model could well explain the observed phenomena.

**Figure 1:**
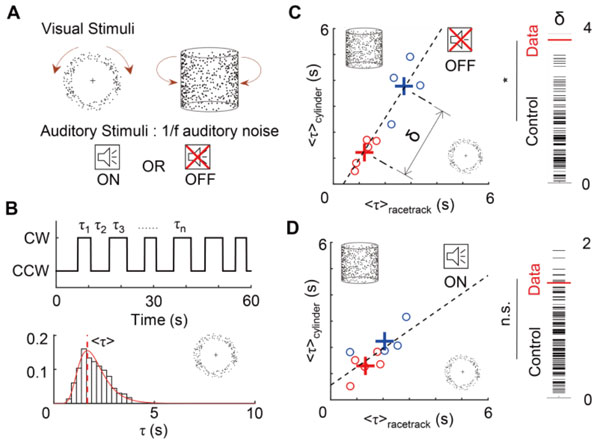
**Effect of auditory noise in visual bistable perception**. A. Two bistable visual movies. B. Example response and τ distribution. C. Peak of τ distribution in no noise condition. D. Peak of τ distribution with auditory noise condition. Auditory noise significantly changed the reversal time and the difference between the two groups has been decreased.

## Conclusions

Our result shows the multi-modal features of bistable perception in visual system and provides evidence of multi-modal integration process in the sensory perception for ambiguous information. We suggest that auditory noise can regulate the temporal switching of bistable perception in neural circuit level.

## References

[B1] Jain S Performance characterization of Watson Ahumada motion detector using random dot rotary motion stimuli PLoS One 2009 4 2 e4536 19225571 10.1371/journal.pone.0004536 PMC2640429

[B2] Kornmeier J Bach M Ambiguous figures - what happens in the brain when perception changes but not the stimulus Front Hum Neurosci 2012 6 51 22461773 10.3389/fnhum.2012.00051 PMC3309967

